# Global Mental Health: Where We Are and Where We Are Going

**DOI:** 10.1007/s11920-023-01426-8

**Published:** 2023-05-31

**Authors:** Modhurima Moitra, Shanise Owens, Maji Hailemariam, Katherine S. Wilson, Augustina Mensa-Kwao, Gloria Gonese, Christine K. Kamamia, Belinda White, Dorraine M. Young, Pamela Y. Collins

**Affiliations:** 1 Department of Psychiatry and Behavioral Sciences, University of Washington, Seattle, WA 98195 USA; 2 Department of Public Health, Department of Obstetrics, Gynecology and Reproductive Biology, Charles Stewart Mott, Michigan State University, East Lansing, USA; 3 Department of Global Health, University of Washington, Seattle, USA; 4 Department of Health Systems and Population Health, University of Washington, Seattle, USA

**Keywords:** Global mental health, Social determinants, Integrated mental health care, Inequities in global mental health

## Abstract

**Purpose of Review:**

To summarize recent findings in global mental health along several domains including socioeconomic determinants, inequities, funding, and inclusion in global mental health research and practice.

**Recent Findings:**

Mental illness continues to disproportionately impact vulnerable populations and treatment coverage continues to be low globally. Advances in integrating mental health care and adopting task-shifting are accompanied by implementation challenges. The mental health impact of recent global events such as the COVID-19 pandemic, geo-political events, and environmental change is likely to persist and require coordinated care approaches for those in need of psychosocial support. Inequities also exist in funding for global mental health and there has been gradual progress in terms of building local capacity for mental health care programs and research. Lastly, there is an increasing effort to include people with lived experiences of mental health in research and policy shaping efforts.

**Summary:**

The field of global mental health will likely continue to be informed by evidence and perspectives originating increasingly from low- and middle-income countries along with ongoing global events and centering of relevant stakeholders.

**Supplementary Information:**

The online version contains supplementary material available at 10.1007/s11920-023-01426-8.

## Introduction

The field of global mental health (GMH) aims to promote mental health, well-being, and treatment for populations across the world, centering transdisciplinary approaches and attainment of mental health equity and human rights [1•, 2]. Over the past 15 years, the field has largely focused on the neglect of mental health and efforts to redress disparities in care between greater and lesser resourced countries [3, 4]. Notably, GMH refers to mental health needs of all countries, focusing on communities at greatest risk for mental health disparities [5, 6]. In 2018, the Lancet Commission on GMH and sustainable development framed the field in terms of four foundational pillars. First, mental health is a global public good. Second, mental health problems exist along a continuum. Third, the mental health of an individual is a unique product of one’s social and environmental influences along with their genetic and biological predisposition. Fourth, mental health is a fundamental human right and requires a rights-based approach [7•]. These domains address priority areas and highlight persisting gaps. Alongside the emphases of the Commission, researchers, activists, and practitioners have sought a greater exploration of the social determinants of GMH, delineating research gaps that encompassed men’s mental health, climate and environmental risks, and the role of spirituality and other sociocultural influences [8•, 9]. With the intensification of social justice movements in 2020, greater attention to mental health equity and structural and political determinants of poor mental health such as discrimination, racism, and poverty have risen in priority [9]. An ongoing critique of GMH includes the emphasis on the application of Western constructs to describe and diagnose mental disorders [9]. The reframed agenda proposed by the Lancet Commission attempts to address some of these limitations by expanding the scope GMH from reducing the treatment gap for mental disorders to improving mental health for whole populations and reducing the global burden of mental disorders [7•]. It also includes ensuring that entities that act as the arbiters of the global mental health field include all voices to achieve these aims. Achieving these aims through research and policy action requires an understanding of the recent global burden of mental disorders, what upstream factors contribute to the onset of mental health problems, advances in mental health care approaches, inequities in GMH treatment and in the context of current global events, trends in GMH funding, and lastly, progress made towards centering relevant stakeholders in GMH research and practice.

This review examines recent advances in global mental health research along several broad domains—current burden of mental disorders, socioeconomic determinants of mental disorders, current priorities in global mental health, funding for global mental health, and progress towards centering important stakeholders in global mental health research and practice.

## Global Epidemiology of Mental and Substance Use Disorders

Mental and substance use disorders are some of the leading causes of disability globally [10, 11]. Depressive and anxiety disorders account for more than 970 million prevalent cases globally in 2019 [10]. The prevalence of substance use disorders has increased substantially since 1990 [10]. Among substance use disorders, alcohol use disorders account for more than 108 million prevalent cases and drug use disorders account for more than 56 million prevalent cases globally. Opioid use disorders are the most prevalent drug use disorder accounting for more than 22% of prevalent drug use disorder cases [11].

According to recent estimates, more than 13% of adolescents globally have a mental disorder, with common mental disorders such as anxiety and depressive disorders comprising about 40% of mental disorders [12]. Mental disorder prevalence continues to show consistent variation by gender with depression and anxiety being more common among females and attention-deficit hyperactivity disorder (ADHD) and conduct disorder being more common among males [10]. The prevalence of substance use disorders also continues to vary by gender with the prevalence in males being twice as high as that of females [11].

Mental disorders not only debilitating but are also risk factors for fatal outcomes such as suicide and all-cause mortality [13–15]. Little is known about the prevalence of and mortality attributed to mental disorders in many low- and middle-income countries due to limited use of representative mental health surveys [16,17•]. While epidemiological research on mental disorders continues to rely primarily on data from high-income countries, more evidence originating from low- and middle-income countries (LMICs) is needed to better understand the true global epidemiology.

## Social and Economic Determinants of Mental Disorders

Social and economic determinants contribute to risk for mental disorders and disproportionately impact populations living in contexts of great adversity [18]. A review of social determinants of mental health aligned with the Sustainable Development Goals (SDG) grouped several risk factors into economic, neighborhood, environmental, and social/cultural domains [19•]. Individual characteristics such as gender, age, and ethnicity are markers of discrimination associated with the early onset of mood, anxiety, developmental, and substance use disorders [19•]. Similarly, economic factors (income insecurity), neighborhood factors (migration, exposure to violence), environmental events (natural hazards, conflict), social factors (parenting, education, social support), and structural factors such as systemic racism are associated with disorders that develop in childhood [19•].

Discrimination is one of the key Sustainable Development Goals (Goal 10) particularly in the context of recent events that have differentially affected members of racialized ethnic groups, sexual and gender minorities, stigmatized religious groups, and other marginalized communities. Multiple national surveys conducted in the USA, South Africa, and other countries have found that experiences of discrimination are associated with an increased odds of developing psychological disorders including depression, anxiety, psychotic disorders, and substance use disorders [20, 21]. In the US context, cumulative exposure to structural and communal discrimination leads to maladaptive coping mechanisms, higher rates of vigilance, and psychological distress among Black Americans [22]. Discrimination along other social hierarchies such as caste and religion in India has resulted in historically disadvantaged groups including Scheduled Castes and Muslims who experience worse self-reported mental health compared to upper caste Hindus [23•]. The study also highlights the need for more nuanced research on dimensions of social inequalities that contribute to poor mental health.

Stigma and discrimination can differentially adversely affect people living with mental health conditions. The Lancet Commission on ending stigma and discrimination in mental health found mental health–related stigma can affect interpersonal relationships, career prospects, and discrimination in health care services received [24•]. Stigma and discrimination are often embedded into structures and institutions that implement laws and practices influencing the lives of people living with mental illnesses. In addition, stigma associated with other individual characteristics, including sexual and gender identities and health conditions, is a risk factor for mental disorders including depression and anxiety [25, 26].

Recent research on risk factors has largely includes appraisals of aggregated evidence and the study of marginalization due to race and ethnicity experienced in high-income countries. More research is needed from other countries where social hierarchies manifest along lines of caste, community, religion, or other subgroups that may contribute to disparities in the onset and treatment of mental disorders.

## Treatment and Care Approaches

An important GMH priority has been to identify and implement appropriate treatment resources for populations in need of mental health care. Treatment approaches to address the continuum of mental health to mental illness range from self-care and informal support to community and facility-based mental health services including innovative digital interventions [27–29]. However, the dearth of trained mental health care providers in most countries is an impediment to the delivery of treatment interventions for moderate and more severe conditions. The global community of mental health researchers and practitioners has contributed to innovations in care to enable greater access to mental health services. In this section, we highlight recent updates in integrated mental health services into primary care and the importance of addressing mental health in HIV care settings. We also explore recent developments in care approaches such as task-shifting.

### Integrated Care

#### Primary Care

Integrating mental health care into primary care settings has been emphasized for many years as an important mode of expanding mental health care access. The Collaborative Care Model (CoCM) developed at the University of Washington is a form of integrated care that treats common mental disorders such as depression or anxiety using a team-based approach trained primary care providers and embedded mental health specialists [30•, 31]. This care model, developed in the context of scarce mental health specialists in the USA, has now been tested in multiple countries including India, South Africa, and Nepal [32–34]. Integrated care has also been shown to reduce stigma around accessing mental health care services at the community level [35•, 36]. However, factors such as organizational readiness, complexity of service user needs, and perceived advantage of interventions can affect the implementation of integrated mental health services within primary care settings [37].

#### HIV

People living with HIV (PLWH) are at an increased risk of experiencing mental disorders, and people with mental and substance use disorders are at greater risk of HIV acquisition [38•]. When HIV and depression co-occur, the risk of adverse outcomes along the HIV care continuum increases through reduced adherence to HIV care and treatment, greater attrition from care, and greater mortality [39]. Evidence-based mental health interventions for PLWH (cognitive behavioral therapy–derived interventions, group support psychotherapy, problem-solving therapy) that also target adherence to care can reduce depressive symptoms and improve HIV outcomes [38•, 40–42]. Communities and countries where HIV is most prevalent often lack access to quality mental health services and though effectiveness trials of integrated interventions have shown some successes, scaling up sustained implementation of integrated HIV and mental care in diverse real world settings remains an area of need [39]. As multilateral organizations like UNAIDS and WHO, alongside funders of HIV prevention, treatment, and care, such as the President’s Emergency Plan for AIDS Relief (PEPFAR) and the Global Fund for AIDS, TB, and Malaria, increasingly advocate for or fund the integration of mental health interventions, more opportunities for implementation and iterative learning emerge. Implementing partners such as the International Training and Education Center for Health (I-TECH) exemplify such opportunities in HIV care settings globally (Table 1).

**Table 1:** Implementing integrated mental health and HIV services: perceived barriers and facilitators in real world implementation

**Perceived barriers and facilitators**	**Case examples**
Risk factors	Lack of family support
	Increased awareness and reduced stigma around using mental health services
Follow-up after screening	Support is needed to ensure follow-up care after initial screening
Staff training	Not enough trained providers
	Supervision continues to be an issue in ensuring that patients are receiving quality mental health care. There is a need for a routine and structured way of assessing provider competencies following trainings
Integrated care	Support needed across overlapping areas: HIV, substance abuse, LGBTQ identity, violence, trauma, depression, etc
	Mental health services that are not clinic-based but community based (like peer support)
	Integration of mental health initiatives where people spend time (churches, schools, community action groups, etc.)
Task-shifting and associated challenges	Consider the importance of social workers in mental health service delivery
	Task-shifting for HIV diagnostic assistants to be trained as retention assistants (for tracing) and psychosocial counsellors to treat mild/moderate mental illness and refer severe cases to psychiatrists
	Overwhelming workload for health care workers
Funding constraints	Lack of available funding despite awareness of importance of mental health
Monitoring and evaluation	Critical need for data around impact of MH/SUD on retention and suppression
	The need to develop a culture of documenting all steps
Need for prioritization of mental health	Mental health care is segregated historically from the rest of the system and integration into HIV care needs to be prioritized by funders like PEPFAR

The International Training and Education Center for Health (I-TECH) at the University of Washington is a global network that aims to develop local partnerships and skilled health care workers (see Supplementary file for more details). Box 1 reports examples of perceived barriers and facilitators as described by I-TECH staff in implementing mental health services in the context of the broader global mental health domains explored in this paper

### Task-shifting

Task-shifting (or task-sharing) uses abbreviated training of health care workers (HCWs) to enable redistribution of tasks for more efficient use of the human resources [43]. It is feasible, acceptable, cost-effective, and can address mental health needs at the community level by supporting early detection, prevention, and care. There is substantial literature on task-shifting as a useful approach to increase mental health care coverage where needed [44]. However, more recent research in the last 3 years has focused on assessing the effectiveness of this approach, challenges involved, and support needed for task-shifting to be successful [45, 46]. Importantly, HCWs being trained in task-shifting need to be appropriately supported (e.g., receive routine supervision) in order to tackle implementation barriers such as stigma and client reluctance to disclose mental health problems [47, 48]. This support may be challenging to provide in most LMIC care settings due to increased workload and limited staff availability. In integrated contexts, lay workers with experience in related areas (e.g., maternal and child care) may find it easier to adopt mental health interventions [47]. There is also recognition of the importance of the role of nurses as they typically take on task-shifting duties, though recent evidence on effectiveness of this strategy is largely derived from HICs [49, 50]. There is also a need for formal protection for task-shifting. The WHO guidance on task-shifting recommends the national endorsement and standardization of registration and career progression pathways for health care workers [51]. However, the absence of a specific taskforce or certification to regulate practice standards for non-professional personnel providing specialized care poses a challenge [52].

Most research on task-shifting was largely encouraging the use of task-shifting as a useful approach to expand mental health care access in low-resource settings. However, in recent years, research on task-shifting has now started to focus more on the nuances and challenges of this approach and is also gradually originating more from LMICs themselves than from HICs.

## Inequities in Access to Care

### Gaps, Progress and Barriers to Treatment

Despite the high prevalence of mental health conditions worldwide, treatment coverage remains low and varies by setting and population. Furthermore, data on treatment coverage is not routinely collected despite it being part of international priorities such as the targets set by the World Health Organization Mental Health Action plan [53]. There are sparse data on treatment coverage from many parts of Sub-Saharan Africa and Asia that comprise nearly three quarters of the world’s population [17•]. This continues to hold true particularly for major depressive disorder. Analyses of treatment coverage data from 84 countries estimated mental health treatment coverage for MDD to be 33% in high-income countries and only 8% in LMICs [17•]. There has been little literature in recent years on treatment coverage for severe mental disorders (SMDs) such as schizophrenia. Recent analyses estimate treatment coverage for schizophrenia to be 9.4% in Ukraine, 19% in the Philippines, and 10% in Ethiopia [54, 55]. According to the 2020 World Mental Health Atlas, many countries likely will not meet WHO/UN targets for mental health care expansion [53]. Barriers to treatment include inadequate financial investment at the government and international levels, bottlenecks to treatment such as inadequate quality of care, and the experience of stigma, fear, and shame surrounding mental illness and suicide [17•, 56].

Progress has been made towards expanding access to treatment using innovative and locally adapted care approaches [57]. Many countries have set up targets and national policies/action plans to address mental health needs [53]. WHO baseline analyses show that early adopter countries all have low treatment coverage and are planning for integrated mental health care and to invest more resources into mental health [54].

### Mental Health Care for Youth

The prevention, diagnosis, and treatment of mental disorders among young people is a global priority [12]. Interventions delivered during adolescence yield a “triple dividend of investment” in terms of youth well-being and health outcomes in adulthood and for the subsequent generations [58]. Mental health interventions for youth delivered in community and school-based settings have been shown to be effective in decreasing mental disorder symptoms and disruptive behaviors as well as improve social skills and personal well-being [59]. The use of digital interventions may also increase engagement in mental health care among children and young people [60]. Additionally, a substantial proportion of young people globally live in urban environments that could increase the risk of developing mental disorders [61]. The provision of safe public spaces and psychological interventions that promote mental well-being and social connectedness are some effective approaches to address urban adolescent mental health [61]. The inclusion of a dedicated goal (SDG 3) promoting mental health and well-being in the Sustainable Development Goals (SDGs) of 2015 marked a notable milestone in the field of youth GMH, as it increased awareness about the importance of mental health among young people and catalyzed actions to enhance mental health care services for youth worldwide [7•]. The Wellcome Trust launched the “Mental Health Priority Area” in 2020 to support the development of novel approaches for preventing and treating mental health disorders in young people worldwide [62]. Mental health interventions for the current generation of youth must also incorporate the adverse impacts of social media exposure such as isolation and loneliness. This was highlighted by the US Surgeon General Dr. Vivek Murthy who emphasized adolescent mental health as a top priority in the wake of increased suicide rates and emergency room visits for self-harm [63].

While much progress has been made in addressing youth GMH, significant gaps remain in on the development of youth mental health interventions particularly in LMICs. Increased investment in mental health services and resources, greater emphasis on mental health literacy and education, enhanced collaboration across sectors, and the development and implementation of evidence-based interventions and treatments are needed to improve youth mental health globally [58].

## Contemporary Priorities for Global Mental Health

We review the literature on four key global events/phenomena that have exacerbated inequities in mental health, namely the current COVID-19 pandemic, political conflict and instability, human rights issues, and environmental events.

### COVID-19 Pandemic

The effect of the global COVID-19 pandemic on mental health has been well described in the literature [64]. A recent GBD paper estimated an additional 53.2 million cases of depression and 76.2 million cases of anxiety due to the COVID-19 pandemic [65]. The pandemic has also been associated with an increased risk of suicidality [66]. The pandemic had a particularly profound impact on the mental health of vulnerable populations with pre-existing conditions that may increase risk of being infected (elderly, chronic conditions) and marginalized communities particularly in LMICs [67]. The pandemic also saw an increased body of research on the mental health of health care workers. Mental health conditions, stress, and burnout were prevalent among health care workers—particularly female health care workers, health care workers from minority groups, and nurses[68]. Caregivers and children were also adversely affected [69].

### Conflict/Instability and Mental Health

International conflict and instability in the last few years have harmed the mental health of displaced populations. A study on the mental health effects of war on children due to the Russian invasion of Ukraine found that the cumulative impact of conflict puts estimated 7.5 million children at extreme mental and physical health risk [70, 71]. According to estimates from WHO, approximately half a million people impacted by this invasion will need mental health treatment [72]. In Ethiopia, young adults in Tigray experienced elevated levels of anxiety and depression due to ongoing conflict [73]. Refugee and migrant host countries will need to address the likely disproportionate number of refugees with PTSD, depression, anxiety, and possible exacerbation of substance use disorders [71]. Recommendations include implementing family-based strengthening interventions and culturally adapted psychological interventions delivered within existing economic platforms such as youth employment and empowerment programs [74, 75]. Future work is needed to assess national priority setting for refugee rehabilitation, and to address long-term impacts of current conflicts particularly on children and vulnerable populations in the context of a near or complete loss of already scarce health care systems [76–79].

### Mental Health and Human Rights Issues

Human rights violations in mental health services are often underreported and underestimated [80]. Several countries have mental health and human rights legislation in place as protective measures [81]. However, it is also important to evaluate the impact and effectiveness of these policies. An evaluation of the United Nations Convention on the Rights of Persons with Disabilities (UN CRPD) implementation in 4 West African countries found several human rights violations and poor standards of living and care for psychiatric inpatients [82]. Well-intentioned legal protection for human rights can have unintended adverse consequences. In Queensland, an evaluation of revised MH legislation found that compulsory treatment orders actually increased after revised legislation despite efforts towards less restrictive treatment [83]. The COVID-19 pandemic added complexity to the problem of human rights protections for those with and even without mental illnesses particularly due to the strict enforcement of quarantine measures and rules [80]. Enforcing lockdown and curfew hours often resulted in human rights abuses perpetrated by law enforcement officers [84, 85]. These findings suggest the possibility of exacerbating and prolonging existing mental illness in the future even in the post-pandemic phase.

### Environmental Events and Mental Health

Environmental and climate change have impacted populations around the world not only due to catastrophic events, but gradual long-term changes as well. This can lead to stress and anxiety particularly for sub-populations that depend on natural resources/natural events for their economic sustenance and risk loss of livelihood and shelter due to environmental events [86, 87]. Research on Nunatsiavut communities in Canada that have historically relied on their lands and environment for their livelihood and traditional practices found higher rates of mental health–related clinic visits after extended periods of warm average temperatures indicating that policy and planning related to climate change should account for mental health implications for Indigenous communities [88]. Climate change has been associated with elevated levels of farmer suicides—a tragically common phenomenon for many years [89–91]. These findings suggest that climate change will exacerbate already existing inequities [92]. Although climate change will likely impact mental health globally, a recent review of climate change and mental health found that 77% of studies came from HICs [87]. This is an important gap since LMICs have been and will likely continue to be severely impacted by climate change. Effective interventions include a focus on recovery, health promotion, resilience, local knowledge, education and awareness of climate change, and improving evacuation procedures [87].

## Funding for Global Mental Health

Robust financing is needed to see strategies through to implementation [93•]. Current funding is not commensurate with mental health needs in many countries and is often hindered by the lack of cohesive policy making efforts and governance [94]. Actual disbursements fall short of pledges and commitments (for all health priorities) [95]. Development assistance for health (DAH) is an important source of global health funding from high-income countries (HICs) for LMICs. In 2019, DAH for NCDs (which includes mental disorders) was $0.7 billion in 2019 which was far lower than the existing target of $28 billion estimated by researchers [96]. A review of OECD data from 2006 to 2016 on development assistance for mental health found that only 0.3% of all official DAH were for projects solely dedicated to mental health. Of leading bilateral donor agencies, only 2 (Austrian Development Agency) and Swiss Agency for Development and Corporation (SDC) mentioned mental health in their priorities [97]. Disparities exist in how funding is allocated for research in GMH. The Inequities in MH funding 2020 Report found that approximately $3.7 billion is spent on MH research grants globally per year mostly from and in HICs [98]. Specific fields such as suicide, eating disorders, and personality disorders are underfunded compared to depression and substance use and dependence. Younger populations are not the focus of mental health research grant funding despite evidence suggesting that most MH conditions occur at younger ages [98]. Most grant funding is funneled towards basic sciences with less than 7% of funding going to prevention [98].

However, progress has been made towards increasing local capacity building for research and sustained mental health services. The Global Alliance for Chronic Diseases consortium of funding agencies selected GMH for its annual call in 2017. A total of US$ 60 million of funding was made available through various participating funding agencies (such as the European Commission, EC; Medical Research Council, MRC; and National Institutes of Health, NIH) with a focus on implementation research [97, 99]. In response to findings from the 2020 NSDUH survey on worsening mental health in children during the pandemic, the US Health and Human Services (HSS) recently announced a $40.22 million grant to address youth mental health in the USA [100].

It is also important to assess what strategies can be adopted to mobilize funds for GMH. An assessment of financing options for mental health in 6 Sub-Saharan showed that including mental health care as part of the national insurance system was an important strategy towards inclusive and equitable funding for mental health financing [101]. A similar assessment of health systems strengthening in Nigeria also identified mental health care integrated into primary care, performance-based financing measures, and engaging with relevant stakeholders and external institutions [102]. In Ethiopia, this assessment identified political commitment, favorable economic growth, and expansion of community-based health insurance as some of the opportunities for improved mental health financing [103].

## Centering Relevant Stakeholders in Global Mental Health

### Lived Experience of Mental Illness

GMH research needs to include and center relevant stakeholders including people with lived experiences of mental illness (PWLE) as their perspectives play a valuable role in shaping interventions and policies for mental health [104, 105]. The WHO Special Initiative on Mental Health aims to include PWLE in their global collaborative networks [106]. The Lancet Commission on stigma emphasizes the need to involve PWLE at all stages of mental health research and the positive impact they can have on mental health programs including facilitating peer-to-peer support, sustained advocacy after program completion, and increased help-seeking [24•]. The Wellcome Trust UK works with a team of PWLE who shape mental health programs with their expertise and insight [107]. These efforts emphasize the importance of treating lived experience expertise at par with other forms of educational and professional expertise [107]. Evidence on research including PWLE although limited at present is rapidly developing and its impact and effectiveness should be evaluated in future studies and reviews.

### Power Dynamics in Global Mental Health Research

There has been increasing attention on GMH methods in LMICs and decolonization of the field. Examples of inequities in research activities typically involve data collection efforts carried out by researchers from HICs in LMICs without acknowledging local research staff and knowledge in scientific publications or engaging in local capacity building—commonly termed “helicopter science” or “extractive science” [108]. These inequities are often the result of research funding being allocated predominantly to institutes in HICs which then exacerbates power imbalances between centers of knowledge in the Global North versus the Global South. Research conducted in LMICs but published by HIC researchers can also impact the analytic choices and interpretation of scientific findings if not adequately grounded in the cultural settings they are derived from. Therefore, important cultural nuances may go unrecognized in published research. Long-term sustainable multi-country research partnerships that engage all contributors equitably and strengthen capacity-building efforts for researchers in LMICs are needed. Most importantly, there is recognition of the need to directly fund local research institutions to enable them to develop their own research programs and provide equitable means of publishing local research in international journals, conferences, and other venues [109].

## Conclusion

**Fig. 1:**
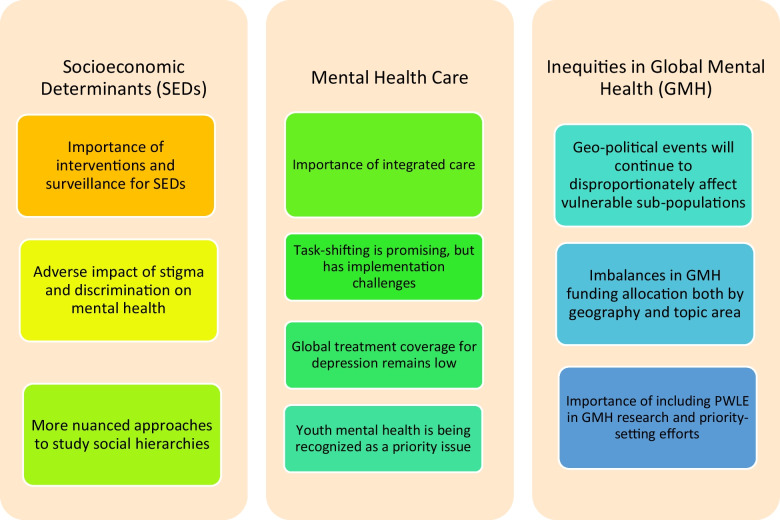
Summary of recent priority areas in the field of global mental health. This figure summarizes recent findings across priority areas in global mental health that are reported in this review

This review highlights recent developments in the field of GMH in the past 3 years. Disparities in mental health conditions and access to adequate mental health care continue to exist globally and disproportionately affect socially disadvantaged populations (Fig. 1). Treatment approaches including integrated care and task-shifting have shown promise but are not without their challenges in implementation. And lastly, there is a growing recognition for the need to include marginalized voices and people with lived experience of mental illness in research and priority-setting efforts. Much progress has been made in the field of GMH though there is an ongoing need to continue promising efforts that are underway. The evolving nature of global events and the centering of relevant stakeholders will likely shape the paradigm of GMH practice and research in the years to come.

## Supplementary Information

Below is the link to the electronic supplementary material.

Supplementary file1 (DOCX 15 KB)
