# Correction to: Global Mental Health: Where We Are and Where We Are Going

**DOI:** 10.1007/s11920-023-01434-8

**Published:** 2023-06-27

**Authors:** Modhurima Moitra, Shanise Owens, Maji Hailemariam, Katherine S. Wilson, Augustina Mensa‑Kwao, Gloria Gonese, Christine K. Kamamia, Belinda White, Dorraine M. Young, Pamela Y. Collins

**Affiliations:** 1 Department of Psychiatry and Behavioral Sciences, University of Washington, Seattle, WA 98195 USA; 2 Department of Health Systems and Population Health, University of Washington, Seattle, USA; 3 Charles Stewart Mott Department of Public Health, Department of Obstetrics, Gynecology and Reproductive Biology, Michigan State University, East Lansing, USA; 4 International Training and Education for Health (I-TECH), Department of Global Health, University of Washington, Seattle, USA; 5 Zimbabwe Technical Assistance, Training and Education for Health (Zim-TTECH), Harare, Zimbabwe; 6 International Training and Education Center for Health (I-TECH), Lilongwe, Malawi; 7 International Training and Education Center for Health (I-TECH), Port of Spain, Trinidad and Tobago; 8 Caribbean Training and Education Center for Health (C-TECH), Kingston, Jamaica

**Correction to: Current Psychiatry Reports** **https://doi.org/10.1007/s11920-023-01426-8**

The original version of this article unfortunately contained mistakes in the authors' affiliations. The corrected affiliations are captured below.

