# Supplementary material for: Global Mental Health: Where We Are and Where We Are Going
Source: Curr Psychiatry Rep. 2023 May 31;25(7):301–11. doi: 10.1007/s11920-023-01426-8 (PMC10230139; doi:10.1007/s11920-023-01426-8)
Supplement: Supplementary file 1 — Supplementary file1 (DOCX 15 KB) [file 11920_2023_1426_MOESM1_ESM.docx]

# Supplementary Information File

Contents:

1. Glossary of Terms used
2. Background on I-TECH at the University of Washington

_____________________________________________________________________

1. Glossary of Terms used:

| Abbreviated Term | Expanded Term |
| --- | --- |
| GMH | Global Mental Health |
| HIV | Human immunodeficiency virus |
| LMIC | Low- and middle-income country |
| HIC | High income country |
| PLWH | People living with HIV |
| PWLE | People with lived experiences of mental illness |

1. Background on I-TECH at the University of Washington:

The University of Washington International Training and Education Center for Health (I-TECH) works with local partners to develop skilled health care workers and strong national health systems in resource-limited countries. The I-TECH Mental Health Learning Network is a multi-country initiative launched in 2022 that aims to facilitate knowledge exchange on mental health care integration within HIV care settings. The learning network meets each month to share lessons and challenges in mental healthcare implementation and build collaborative partnerships among country teams in Asia, Africa, the Caribbean, and the United States. The excerpts provided in this paper are shared by members of the learning network at I-TECH who draw on experiences with integrating mental health care into HIV settings and training healthcare workers in providing mental health care to people living with HIV.
